# Genital pearling: How many can fit? A case report

**DOI:** 10.1016/j.eucr.2026.103436

**Published:** 2026-04-07

**Authors:** Muhammad Rafid Murfi, Kuncoro Adi

**Affiliations:** a Department of Surgery, Division of Urology, Universitas Padjajaran, Bandung, Indonesia; b Department of Surgery, Division of Urology, Hasan Sadikin Hospital, Bandung, Indonesia

**Keywords:** Penile pearling, Genital beading, Penile implants, Sexual dysfunction

## Abstract

Penile pearling, or subcutaneous penile implantation, involves inserting small objects beneath the penile skin to enhance sexual pleasure or as a cultural symbol. We report a rare case of a 63-year-old man who presented with a painful scrotal lump and fever. Further evaluation revealed a history of self-inserting 90 subcutaneous penile beads ("pearls") using modified toothbrush handles. Surgical removal was performed using penile degloving technique under regional anesthesia, successfully extracting all 90 implants with uneventful recovery and preserved erectile function at one-month follow-up. This case underscores the need for early recognition and proper management of complications related to penile pearling.

## Highlights

• A rare case of extreme penile pearling with a total of 90 subcutaneous implants is reported.
• Penile degloving technique enabled complete removal of multiple implants in a single procedure.
• Surgical removal preserved erectile and urinary function with favorable short-term outcomes.
• Early recognition and culturally sensitive management are essential in penile pearling cases.

## 1 Introduction

Penile pearling, or subcutaneous penile implantation, is a form of body modification where small objects such as beads or pearls are inserted beneath the skin of the penis.^1^ **It is often performed to enhance sexual pleasure or as a symbol of social identity across various countries****.**^2^^,^^3^ Despite its **recognized practice**, penile pearling is associated with various complications, including infection, **tissue injury**, sexual dysfunction, and increased risk of sexually transmitted infections (STIs).^4^^,^^5^

While many cases remain asymptomatic, the procedure is frequently performed under non-sterile conditions, increasing the risk of complications. Surgical removal is often required when implants cause pain, inflammation, or functional impairment. In this report, we present a rare case involving the discovery of 90 penile pearls **managed using a penile degloving technique, highlighting the surgical challenges and management of strategy for extensive penile pearling.**

## 2 Case presentation

**Fig. 1:**
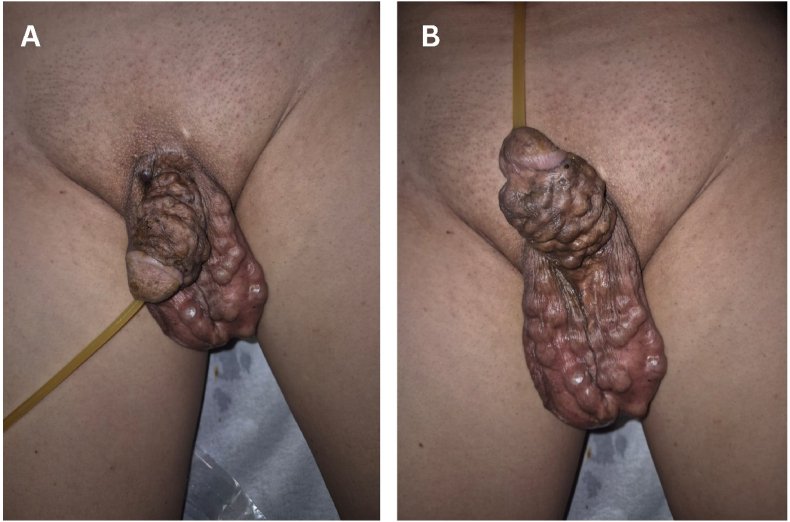
Clinical presentation of the patient. (**A**) dorsal aspect, (**B**) ventral aspect.

A 63-year-old male presented with a chief complaint **of a painful lump on the left side of the scrotum accompanied by fever. The patient reported that the lump increased in size over time.** The patient denied any associated nausea or vomiting. The patient disclosed a history of inserting "beads" (locally referred to as "tasbih") into the penile shaft at the age of 17. The beads were crafted from the handle of a toothbrush, shaped into round or oval forms. During each session, one or two beads were inserted into the penile shaft with the assistance of a friend (Fig. 1). The procedure involved stretching the penis, identifying the absence of major blood vessels, making a small incision on the penile skin using scissors, inserting the beads, and then closing the wound with povidone iodine plaster for approximately three days until spontaneous healing occurred. The patient did not experience any urinary or sexual dysfunction during or after the bead insertion period.

His medical history was significant for hypertension but negative for diabetes mellitus, heart disease, and kidney disease. He was taking amlodipine 5 mg daily for hypertension. His social history revealed that he smoked approximately eight cigarettes per day and consumed alcohol. There was no history of previous surgeries.

**Fig. 2:**
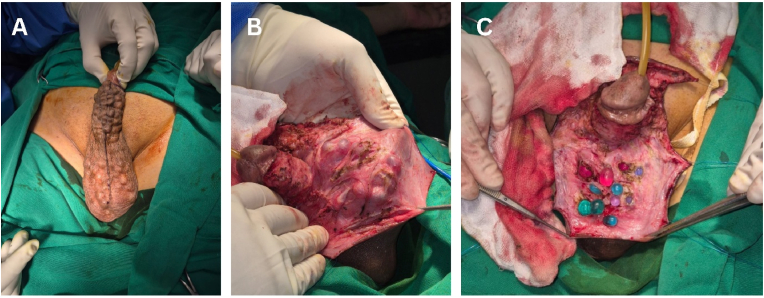
Intraoperative findings. (**A**) before incision and degloving, (**B**) penile degloving exposing subcutaneous pearls, (**C**) extraction of embedded pearls.

**Fig. 3:**
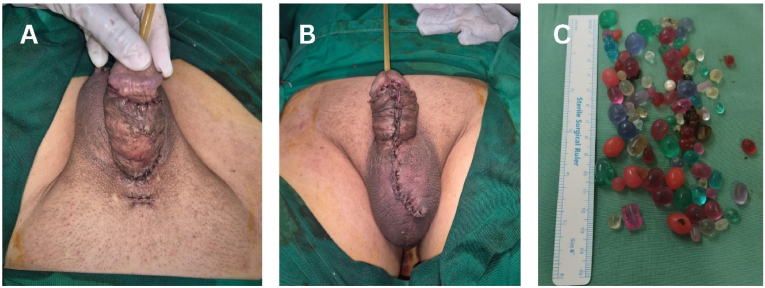
Post-operative condition of the patient. (**A**) dorsal aspect, (**B**) ventral aspect, (**C**) extracted penile pearls.

In this case, the surgical removal of the penile pearls was performed using a penile degloving technique **under regional anesthesia** (Fig. 2). A circumferential subcoronal incision was made, and the penile skin was carefully degloved to expose the subcutaneous tissue and implants. The pearls, which were distributed along the shaft, were meticulously extracted one by one using blunt dissection. Hemostasis was achieved, and the wound was sutured with absorbable material (Fig. 3). The patient was given prophylactic antibiotics and advised on wound care. Postoperative recovery was uneventful, at one-month follow-up after the intervention, the patient reported no urinary complaints. Erectile function was preserved with an Erection Hardness Score (EHS) of 4. **No wound complications, including infection or skin necrosis, were observed.**

## 3 Discussion

Penile pearling, is a body modification involving the insertion of small objects such as beads, pearls, or plastic pieces beneath the skin of the penis.^6^^,^^7^ This practice has a long and complex history, with deep cultural, social, and sexual significance. It is particularly common among prisoners, drug users, sailors, and certain subcultures in Asia, Eastern Europe, and the Americas.^7^, ^8^, ^9^, ^10^ **The materials is include plastic form, metal, or even organic materials**.^8^^,^^11^ The motivations behind penile pearling are diverse and complex. The increased friction caused by the beads during intercourse is thought to heighten sexual stimulation.^9^ Aside from sexual gratification, pearling is also driven by social and cultural factors. Among prisoners, pearling symbolizes resilience and toughness.^8^^,^^11^

Patients with penile pearls often present asymptomatically, with the implants being discovered incidentally during physical examination or imaging. However, symptomatic cases may present with (1) pain or discomfort during sexual activity; (2) erythema, swelling, or ulceration of the penile skin; (3) signs of local or systemic infection (e.g., purulent discharge, fever); (4) dyspareunia for sexual partners; and (5) spontaneous extrusion of the beads.^5^, ^6^, ^7^, ^8^^,^^12^, ^13^, ^14^, ^15^, ^16^.

**The present case is for the extreme of 90 pearls, which raises significant concerns regarding the risk of structural damage and vascular compromise.** The sheer number of implants increases the risk of mechanical injury to the penile tissue, including damage to the tunica albuginea, corpora cavernosa, and urethra. Improper placement of the beads can cause erectile dysfunction, penile curvature, and urethral fistula formation.^5^ The mechanical stress placed on the penile tissue by the implants may also lead to vascular insufficiency and necrosis. Furthermore, the presence of multiple implants increases the likelihood of spontaneous extrusion, where the body rejects the foreign object and attempts to expel it through the skin, resulting in chronic scarring and tissue loss.^5^^,^^16^

Infection is one of the most common complications associated with penile pearling, particularly when the procedure is performed in non-sterile environments.^7^^,^^11^^,^^17^ Infections are often caused by *Staphylococcus aureus* and anaerobic bacteria, necessitating broad-spectrum antibiotic therapy.^5^ In severe cases, surgical drainage of abscesses and removal of the infected implants are required.

Sexually transmitted infections (STIs) are another significant concern associated with pearling. The presence of multiple beads increases the likelihood of microtears in the penile mucosa during intercourse, creating entry points for pathogens.^13^^,^^17^

Structural damage resulting from improper placement of implants can lead to long-term sexual dysfunction.^5^^,^^11^^,^^16^ Urethral injury may lead to fistula formation and urinary incontinence.^4^

Management of penile pearling is primarily determined by the presence or absence of complications, including infection, structural damage, sexual dysfunction, and psychological distress.^5^ A study by Ramirez et al. emphasized that do not typically require intervention unless the patient desires removal for personal or psychological reasons​; **however, surgical removal is indicated in the presence of infection, pain, or functional impairment. Conventional removal techniques typically involve multiple small incisions over each implant**.^12^

Surgical removal of penile implants is indicated in cases of infection, structural damage, or patient distress.^5^^,^^6^^,^^8^^,^^14^^,^^16^ **Conventional techniques involve multiple small incisions over each implant.** In this case, the unprecedented number of 90 subcutaneous penile implants necessitated a more extensive surgical approach and a penile degloving technique was chosen for its ability to provide wide exposure, minimize tissue trauma, and ensure complete removal of all implants. This technique involves making a circumferential incision at the subcoronal level and mobilizing the penile skin proximally to expose the entire shaft. The approach allows for a more efficient removal of multiple foreign bodies while preserving neurovascular structures and maintaining cosmetic and functional outcomes. The degloving method offers several advantages in mass implant removal, such as facilitating better visualization and assessment of underlying tissue damage, reducing the risk of missed implants or repeated incisions, and allowing for uniform irrigation and infection control.

Importantly, no postoperative complications, including infection or skin necrosis, were observed, and erectile function was preserved. This suggests that, when performed carefully, the degloving technique is both safe and effective even in extreme cases. This case underscores the importance of appropriate surgical planning and a patient-centered approach in managing extreme penile pearling.

## 4 Conclusion

The case of 90 pearls underscores the need for a balanced and culturally sensitive approach to managing penile pearling. While the practice may seem extreme or dangerous from a clinical perspective, it holds deep personal and cultural significance for many individuals. Healthcare providers should adopt a non-judgmental and patient-centered approach, recognizing the social and psychological factors that drive pearling. Education and harm reduction strategies, including proper wound care, STI prevention, and counseling on safe sexual practices, are essential in minimizing the health risks associated with penile pearling.

## CRediT authorship contribution statement

**Muhammad Rafid Murfi:** Writing – review & editing, Writing – original draft, Visualization, Project administration, Methodology, Investigation, Funding acquisition, Formal analysis, Data curation, Conceptualization. **Kuncoro Adi:** Writing – review & editing, Validation, Supervision, Resources, Project administration, Methodology, Investigation, Funding acquisition, Formal analysis, Data curation, Conceptualization.
